# Gold

**Published:** 1875-05

**Authors:** G. V. Black

**Affiliations:** Jacksonville, Illinois.


### SELECTED ARTICLES.

---

### ARTICLE V.

### *Gold.*

BY G. V. BLACK, JACKSONVILLE, ILLINOIS.

*Gentlemen of the New York Odontological Society:*

Your committee have seen fit to invite me to prepare a paper for your Society, naming as the subject, "Gold." While I have an ever-wakeful interest in the welfare and progress of my chosen profession, in the study and practice of which I am spending the best energies of my life, and am always willing to contribute my mite when called upon, I cannot but feel that the committee, in making their choice, have passed by very many men who are far better able both to interest you and benefit the profession. I will nevertheless attempt to say something of the laws governing the welding of metals, which I hope may not be without interest and substantial benefit to some of you in the development and management of that wonderful property in gold, as we use it in our daily practice.

Leaving the history of the development of our understanding and use of this property of gold to those who have preceded me, I will simply remind you that the great mass of dentists now in practice depend upon the welding property of their gold for the permanence of their work. This though is sufficient to show that too great an importance cannot well be attached to its study. In this study we have to deal, as you will presently see, with the most subtle and hidden chemical affinities known to science; and in order that we may start at the best point to gain an understanding of these forces, we will refer to some familiar experiments.

If a piece of fine platinum sponge be prepared, strongly annealed, and allowed to cool, and then held in a jet of hydrogen gas, it will be heated to redness, and the gas ignited. This result is explained in this wise. When the platinum sponge is held in the jet of hydrogen, the gas condenses upon the metal and within its pores with such great rapidity that, its latent heat becoming sensible heat, the sponge is heated to incandescense and the gas ignited.

Again, if we introduce the prepared sponge into a mixture of hydrogen and oxygen gases, the mixture will be exploded; but if the spongy platinum be mixed with some substance to modify the rapidity of the condensation its pores will fill with water. In this case both the hydrogen and oxygen are strongly attracted to the metal; and in condensing upon it, their affinities are so excited that they combine and form the water that fills the sponge. In neither of these instances does either of these elements unite with the platinum, or change it in any degree, while they cannot unaided form a chemical union with the platinum; yet there is a subtle affinity existing between them which causes these elements to condense upon the surface of the metal, and adhere with great tenacity until this attraction is overcome by a powerful heat. By a long course of experiments we have shown that similar phenomena, but less marked, take place with all the noble metals, and are almost certainly found with metals of the baser sort, as a preparatory step to the formation of oxides, etc.; the metals differing from each other in attracting this gas, or that, more strongly than another.

In order to prove that this is true of gold, roll several sheets of pure gold foil into a loose rope, anneal, and suspend them in sulphureted hydrogen gas for a few hours, after which suspend them in ammoniacal gas, or drop them in the liquid ammonia, and carefully dry without heat. Now re-anneal this rope, holding a cold porcelain slab directly over it, and a sublimate will be collected, which proves to be sulphuret of ammonium. If this experiment be varied by substituting chlorine for the sulphureted hydrogen, a much larger amount of sublimate will be collected, which is the chloride of ammonium. This case is chemically identical with the experiment with the platinum in which the water is formed, the only difference being in the elements employed. I might give you a greater variety of these interesting experiments with gold, and different gaseous elements, or with other metals, but, as our space must be limited, this must suffice as illustrating the laws of this exceedingly subtle affinity, which we have found to be as varied and complex as the more prominent and complete affinities of the elements.

Now, I wish to draw your attention to a remarkable circumstance that unerringly accompanies the last and similar experiments. When the rope of gold was annealed preparatory to being placed in the sulphureted hydrogen gas, its welding property was developed, and it might be welded perfectly and solidly together; but when removed from that gas, that property has entirely disappeared. It will not weld any more than so much tissue-paper. The same thing happens if the gold be placed in a bath of ammoniacal gas, hydrogen, illuminating gas, phosphorated hydrogen, sulphurous acid gas, and a great variety of others,—in fact in any gas that is attracted to and condensed upon gold, the effect being more or less perfect according to the power of this peculiar affinity in any particular case.

Now, why the loss of the welding property? The answer is simple—the gas is condensed upon, and covers the surface of the metal, and prevents its particles from coming into actual contact, or within the sphere of their mutual attraction. It cannot be seen by the mortal eye, nor be detected by the ordinary tests for the purity of the metal, but such experiments as those given above reveal it unerringly, and render it appreciable to our senses. After close experimental study of these phenomena with gold and other metals, we believe ourselves warranted in saying that all metals are welding metals, whether cold or hot, if the particles come in contact. Therefore, any metal that is capable of being made actually clean, its surface free from combinations with or condensations of other substance, will readily weld. In other words, if the particles of a given metal are brought into contact, they cohere. This is the secret of the success of using fluxes in soldering and welding by heat. By using a substance that will dissolve and remove oxides formed, and exclude that element, yet adhere to the metal with but little tenacity, so that it is easily thrust aside by the blows of the hammer or the superior affinity of the flowing metal, the particles come into contact, and union results. It also explains those cases of accidental cold or nearly cold welding, causing the sudden stoppage of machinery, especially heavy stones, buggy-wheels running upon iron axles, and so forth. I have examined a number of these cases where a very perfectly weld had taken place over a considerable portion of the surfaces in contact. The attrition had worn away the prominences, and perfectly cleaned the surfaces, until a considerable extent of surface had come into actual contact, and a weld was the result.

I have also been able to produce this result in the machine shop, and thus study its phenomena. This mode of cold welding with iron and steel is, however, entirely impracticable for ordinary mechanical purposes, and this must evidently be the case with any metal that condenses oxygen, for the reason that that element is always free in our atmosphere. This property of condensing oxygen effectually prevents our using the welding property of platinum foil for the purpose of filling teeth, for it requires a white heat to clean it perfectly, and before it is cold the oxygen of the air begins again to condense upon it, obscuring its welding property. If, however, the platinum foil should be covered with ever so thin a lamnia of gold but sufficient to protect it from oxygen, I have no doubt but that it may be readily welded, though I have never tried it. It would probably be necessary to work quite rapidly, so that the surface of the platinum should not be long exposed to the air; otherwise the weld would certainly be lost. The secret of the welding of gold, cold, lies in the fact that it does not attract or condense oxygen; and nitrogen (the other element of our atmosphere, which some poetical chemist has characterized as "that slothful element that never knew a passion, neither of hate, nor yet of love") is, I believe, not attracted by any of the metals. Therefore, gold does not loose its welding property in pure air, or if so, it is only after many days, and then, we believe it is always from the condensation of carbonic acid gas, which is always present in small quantities, and is attracted to gold quite feebly, but with sufficient power to remove the welding property promptly if placed in the pure gas. Therefore, gold left exposed to the ordinary atmosphere loses this property in a few days; but it may be retained for a great while by very close wrapping. The effect of annealing, so far as the development of the welding property is concerned, is simply the cleansing of its surface by driving off any of the volatile gases that may be condensed upon it. This is a very different effect from that of softening gold plate by annealing. The softened plate is not again hardened by exposure to the air, or any of its impurities, while the welding property is so lost.

The welding property is not necessarily lost by hammering, or even burnishing, while its softness is inevitably lost.

Therefore, these two effects of heat are entirely different in their nature; the one cleans the surface, while the other probably causes a repolarization of the atoms. I should probably state that, by hammering gold, the power of the weld is weakened in proportion to the weakening of the general cohesion of the atoms of the mass. It is well known to workers in gold that it may be hammered till it becomes very frail and brittle; and its particles, having lost partially their cohesion or attraction for each other, will of course have lost in the same degree their attraction for fresh masses, and the weld will be weakened to this extent. It is therefore injurious to continue malleting our fillings after all spaces are closed.

These gases, in escaping from our gold in the flame of the annealing lamp, often cause a peculiar appearance which most of you, no doubt, have occasionally noticed. I have often heard operators say that their gold smoked, or that it colored the flame of a lamp. The escape of some of the gaseous elements, and combinations in a pure alcohol flame, are extremely beautiful, among which chlorine deserves especial mention. These phenomena should be observed in a dark room We have so far spoken of these condensations as being readily driven off by heat, after which the welding property is completely restored. With gold, this is true of all but two groups, those formed from sulphur and phosphorous. Any gas into which either of these elements enters is liable to become so rigidly attached to the gold that it cannot be perfetly removed by annealing, and the weldering property of the foil cannot be redeveloped. Therefore, if we would keep our gold in condition to use the welding property, it must be protected from these elements in all their forms. Simple close wrapping does not always suffice, for I have known a number of instances of gold being ruined when quite closely wrapped; and, again, it may be much injured while being prepared for use, or even while on the operators tray; for it sometimes happens that these results are produced quite suddenly.

Though, as these gases are usually found diluted in the air, it generally requires some time for them to become so firmly fixed as to make the lss of the welding property irreparable, yet every operator should be (as I suppose most of us are) especially careful that these gases are not generated in or about his office, from decaying blood in a neglected spittoon, decaying flesh, old teeth, badly arranged sewer connections, etc.; in a word, he who handles gold should be *clean*. Especially would we warn operators to have a care in the use of friction-matches; for every match that is lighted evolves both sulphur and phosphorous fumes, and gold is often injured by them, even when its welding property is not wholly destroyed; especially is this the case when a small portion of phosphorous happens to be detached from the match and lodged upon the wick of an alcohol lamp, which being surrounded by the flame, does not at once burn, but slowly exhales its fumes to light upon the gold as it is being annealed; for the fumes of this element will attach themselves to gold when it is very hot, especially when just taking the form of phosphoric acid, which is not volatile at the annealing heat. That the gold may be injured so as to endanger our work when the welding property is not entirely destroyed, we can probably make clearer by referring to some things that are more patent to the eye.

You have doubtless all noticed that, when a bright surface of any metal is corroded, it almost uniformly happens that the first traces of rust appear at small points, which gradually spread until the whole surface is covered. Now, just the same thing happens in the condensation of the gaseous elements upon the metals, and thus it happens that the welding property is first lost in little spots, while the rest of the surface will weld. This may be proven by close examination of the gold itself as to the welding at different small points of surface; and also by closely watching it during the condensation of those elements that discolor it. This phenomenon may be seen by exposing the gold to ordinary illuminating gas, or others of the carbon group. This is sufficient to show us that when these condensations take place slowly they tend to radiate from centers, which, in some degree, remind us of the points of commencing crystallization. This phenomenon is the one of great danger to us in using gold in filling teeth, for if the welding property is entirely removed, we will not use it; but if most of the surface is in fair condition, we are apt to proceed, though we may even notice that some points do not adhere well, and thus turn off fillings that are imperfect, perhaps to mortify us in a few months or years with their rough surfaces and ragged margins. If gold is to be welded at all, it should be perfectly welded, so that if we take a sharp chisel and pare off a shaving, it will not break or crumble, but will curl up as one pared from the ingot, which is always easy if the foil be in perfect condition. If it is to be used soft, let it be actually non-welding, and each portion be so placed as to be held in position by another portion by wedging, as we used to do in years gone by; for then we know what we do, and can depend upon the result, while, if we attempt to make it weld, and fail, disaster must follow.

Now, as to the protection of gold from these evil influences,—close care in keeping it well excluded from the air is very generally successful, and the effect of such care has been long understood, and practiced with good results. Notwithstanding this, the remark, that "my gold is not working first rate to-day," is often heard, and it has long been understood that atmospheric changes had something to do in the matter. Just what those atmospheric changes may be which cause our gold to work badly to-day, and, passing away allow it to work well to-morrow, is not yet fully understood; for the condensations of which we have spoken do not pass off of themselves. True, the effects of acid compounds are neutralized by alkaline compounds; the affinities in this case being the stronger, the attraction to gold is overcome, which accounts for many changes in condition; yet, in my experiments of leaving gold in the open air in different changes of weather, I find it difficult to explain all the variations of condition in this way, and there are grounds for supposing that ozone may have something to do with it; but this peculiar substance—or state of oxygen, if you prefer the phrase—is very difficult to experiment with, and my efforts have not been satisfactory. It seems true, however, that ammonia will protect gold from all those gases into which those elements which produce permanent injury enter; partly, perhaps, by its previous occupancy of its surface, but principally by combining with them to form harmless salts. Therefore, so long as the gold drawer is kept scented with ammonia, the gold is safe from all harm, and will work each day equally well, no matter what the weather or condition of the atmosphere. This fact I have had occasion to put to the severest practical tests. About eight years ago I arranged a chemical laboratory adjacent to my operating-room; classes recited on certain evenings, and a great variety of experiments were performed for their benefit. Every now and then I found that the welding property of my gold had disappeared, and finally suspected that fumes from this laboratory had some connection with the mischief. I set about an investigation. And a few experiments in exposing gold to this gas, and that with effects that surprised me at every step, awakened an interest in the subject which kept my spare hours engaged for many months, which developed the facts and theories given here, and in a paper presented to my own State Society in the year 1869, and published in the July number of the *Missouri Dental Journal* of that year; also republished in the *Dental Times*, and *American Journal of Dental Science*, somewhat later in the year, to which those wishing details in regard to performing these experiments are referred. Now, after this investigation, classes continued their recitations as before. My gold-drawer was kept scented with ammonia, and no more gold was injured. We had discovered the true source of trouble, and a successful remedy that has never yet failed us. This ammoniated gold is also, in my opinion, a most excellent soft gold. It is perfectly non-adhesive, and for a number of years I have used it for packing against frail margins, and other points where I desired perfectly non-adhesive foil, and find it works admirably and with perfect certainty. The ammonia is perfectly driven off by a moderate heat, and the gold thus becomes perfectly cohesive. By this method of manipulation, we have our gold non-adhesive or adhesive at will. I have observed very closely to discover any objection to this use of ammoniated gold, but have found none in six years' use of it. Besides this, all non-adhesive gold is made non-adhesive by chemical manipulation, and I prefer to know what I am using, though I do not pretend to say that other non-adhesive foils may not be in every respect as good as the ammoniated. I had intended to say something of the liabilities of failure in making perfectly tight margins with cohesive gold, but my paper is too long already. I will therefore close, regretting very much that I am not, at this time, able to be with you.—*Transactions of the New York Odontological Society.*

---

